# 1018. Bacterial Bioburden Characterization of Infected Diabetic Foot Ulcers in Hospitalized Patients in Association with Clinical Outcomes: Traditional Cultures vs. Molecular Sequencing Methods

**DOI:** 10.1093/ofid/ofab466.1212

**Published:** 2021-12-04

**Authors:** Hadar Mudrik-Zohar, Shaqed Carasso, Tal Gefen, Anat Zalmanovich, Michal Katzir, Yael Cohen, Yossi Paitan, Naama Geva-Zatorsky, Michal Chowers

**Affiliations:** 1 Meir Medical Center, Israel, Kadima-Tzoran, HaMerkaz, Israel; 2 Technion – Israel Institute of Technology, Haifa, Hefa, Israel; 3 Meir Medical Center, Kfar- Saba, HaMerkaz, Israel; 4 Meir Medical Center, Tel-Aviv University, Israel, Kfar-Saba, HaMerkaz, Israel; 5 Technion – Israel Institute of Technology, Israel, Canadian Institute for Advanced Research (CIFAR) , Canada, Haifa, Hefa, Israel

## Abstract

**Background:**

Infected diabetic foot ulcers (IDFU) are a major complication of diabetes mellitus. These potentially limb-threatening ulcers are challenging to treat due to the impairment of wound healing in diabetic patients and the complex microbial environment characterizing these ulcers. Our aim was to analyze the microbiome of IDFU in association with clinical outcomes.

**Methods:**

Wound biopsies from IDFU were obtained from hospitalized patients and were analyzed using traditional microbiology cultures, 16S rRNA sequencing and shotgun metagenomic sequencing. Patients’ characteristics, culture-based results and sequencing data were analyzed in association with clinical outcomes.

Study Design

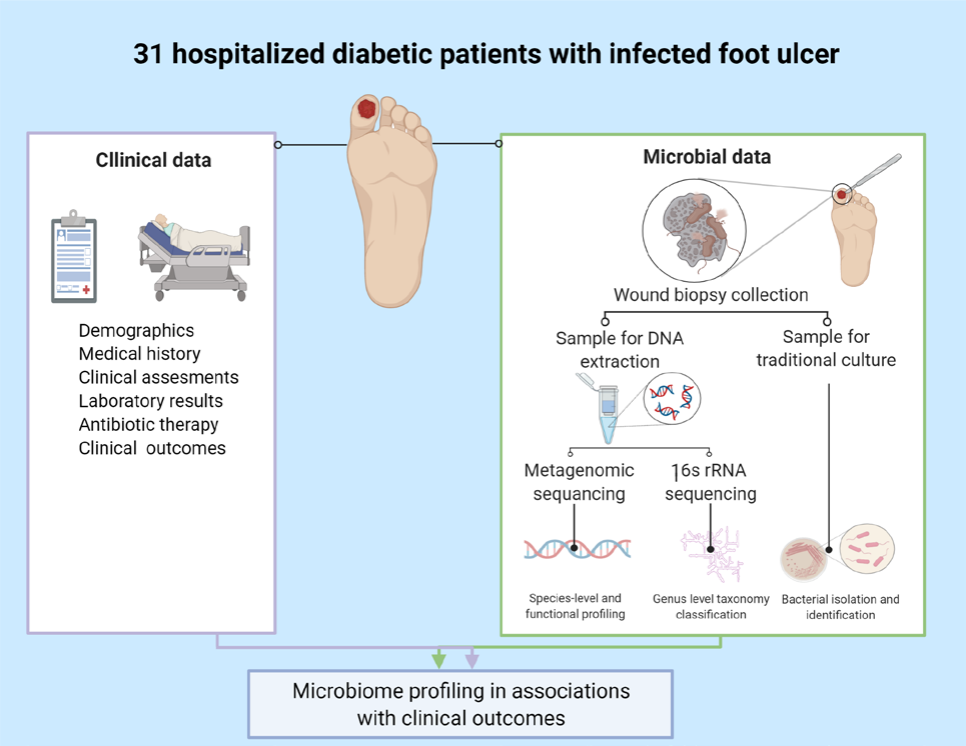

**Results:**

31 patients were enrolled. Significantly more anaerobic and Gram-negative bacteria were detected with sequencing methods compared to conventional cultures (59% and 76% were anaerobes according to 16SrRNA and metagenomic respectively vs. 26% in cultures, p=0.001, and 79%, 59% and 54% were Gram negative bacteria respectively, p< 0.001). Culture-based results showed that *Staphylococcus aureus* was more prevalent among patients who were conservatively treated (p=0.048). In metagenomic analysis the *Bacteroides* genus was more prevalent among patients who underwent toe amputation (p< 0.001). Analysis of metagenomic-based functional data showed that antibiotic resistance genes and genes related to biofilm production and to bacterial virulent factors were more prevalent in IDFU that resulted in toe amputation (p< 0.001).

Occurrences and mean relative abundances of the most prevalent bacteria of IDFU

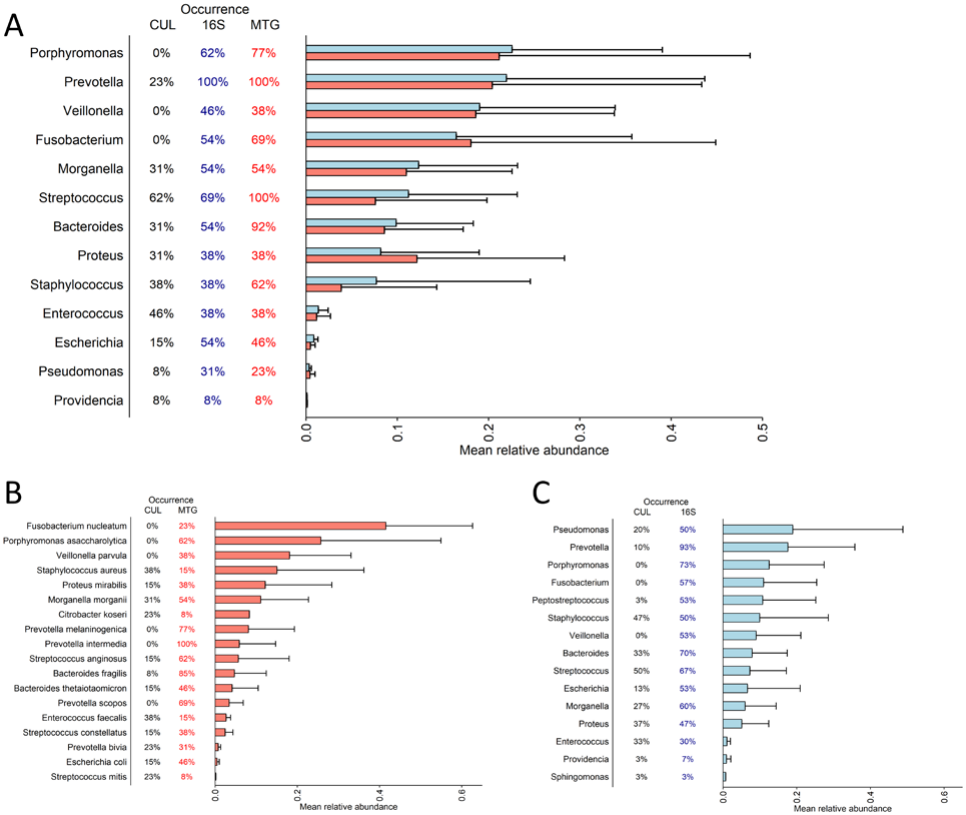

Comparison between [A] traditional cultures, 16S rRNA sequencing and metagenomic sequencing results (genera level - 12 samples) [B] traditional cultures and metagenomic sequencing results (species level – 30 samples) [C] traditional cultures and 16S rRNA sequencing results (genera level - 30 samples) CUL – cultures; 16S - 16S rRNA sequencing; MTG – metagenomic sequencing

Bacteroides genus association with toe amputation

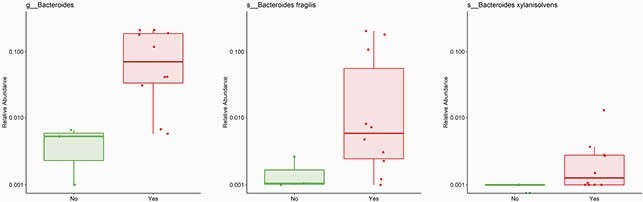

Bacteroides genera was more common among samples of patients who underwent toe amputation compared with samples of patients who were conservatively treated (p < 0.001). Species level analysis showed that Bacteroides fragilis and Bacteroides xylanisolvens predominated IDFU of patients who underwent toe amputation (p=0.04, p=0.002 respectively). No – conservative treatment; Yes – toe amputation.

Functional genes differentiating patients who underwent toe amputation from conservatively treated

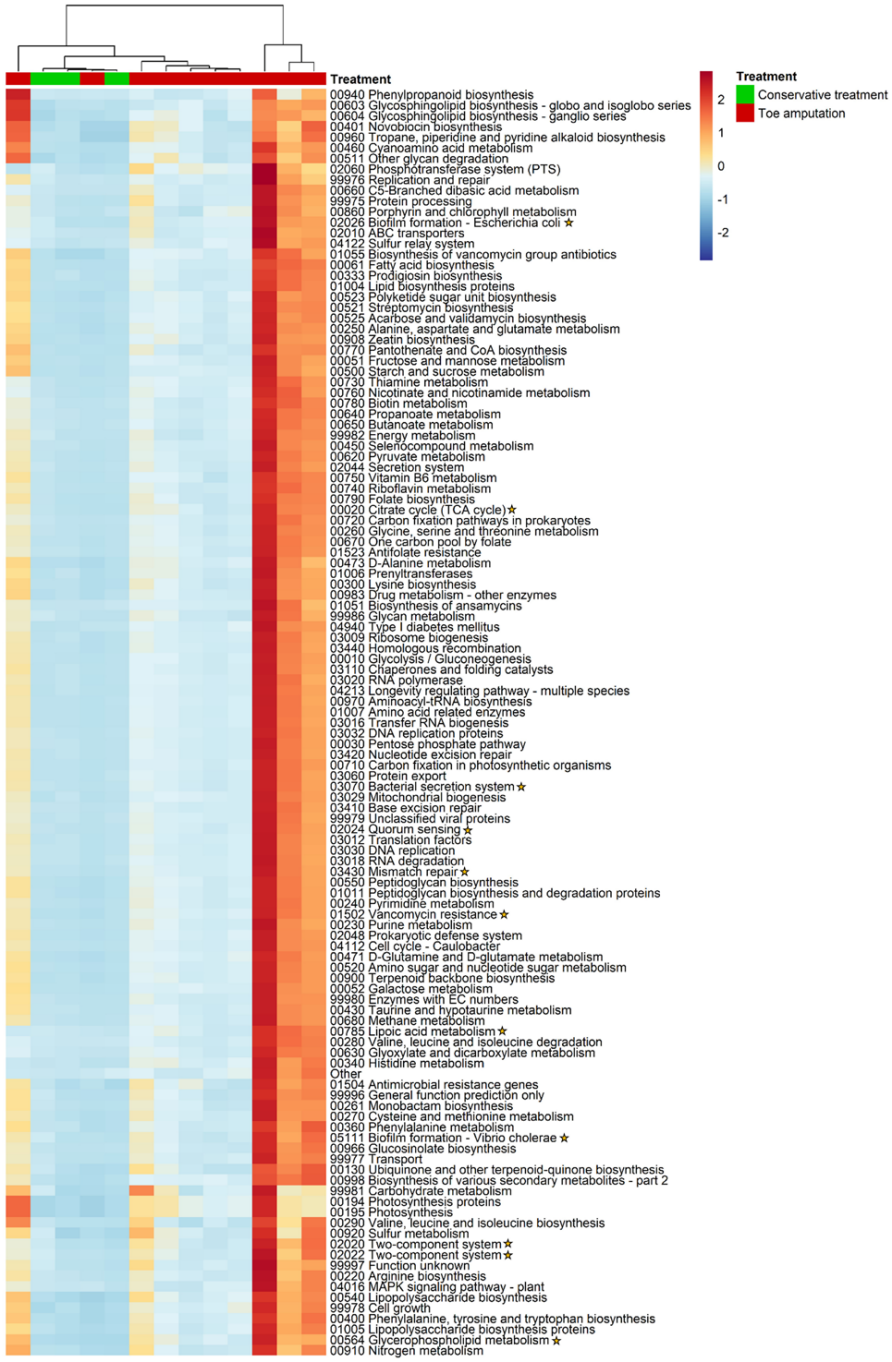

Yellow stars – indicate genes that were associated with bacterial virulent factors, biofilm formation and resistant mechanisms – all were more prevalent in patients who underwent toe amputation (with p values<>

**Conclusion:**

Molecular sequencing tools uncover the complex biodiversity of IDFU and emphasize the high prevalence of anaerobes and Gram-negative bacteria in these ulcers. Furthermore, sequencing results highlighted the possible association between certain genera, species, and bacterial functional genes to clinical outcomes

**Disclosures:**

**Yossi Paitan, PhD**, **Ilex Medical Ltd** (Employee, Other Financial or Material Support, As of 01.01.2021 I am the Laboratories Manager of Ilex Labs)

